# Monitoring of Ex Vivo Cyclosporin a Activity in Healthy Volunteers Using T Cell Function Assays in Relation to Whole Blood and Cellular Pharmacokinetics

**DOI:** 10.3390/pharmaceutics14091958

**Published:** 2022-09-16

**Authors:** Aliede E. in ’t Veld, Manon A. A. Jansen, Bertine W. Huisman, Mascha Schoonakker, Marieke L. de Kam, Dirk Jan A. R. Moes, Mariëtte I. E. van Poelgeest, Jacobus Burggraaf, Matthijs Moerland

**Affiliations:** 1 Centre for Human Drug Research, 2333 CL Leiden, The Netherlands; 2 Department of Surgery, Leiden University Medical Center, 2333 ZA Leiden, The Netherlands; 3 Department of Gynaecology, Leiden University Medical Center, 2333 ZA Leiden, The Netherlands; 4 Department of Clinical Pharmacy & Toxicology, Leiden University Medical Center, 2333 ZA Leiden, The Netherlands; 5 Leiden Academic Centre of Drug Research, 2333 AL Leiden, The Netherlands

**Keywords:** therapeutic drug monitoring, TDM, immunomonitoring, cyclosporine A, CsA, calcineurin inhibitors, CNI, pharmacodynamic

## Abstract

Therapeutic drug monitoring (TDM) of calcineurin inhibitors (i.e., tacrolimus and cyclosporin A) is standard of care after solid organ transplantation. Although the incidence of acute rejection has strongly decreased, there are still many patients who experience severe side effects or rejection after long-term treatment. In this healthy volunteer study we therefore aimed to identify biomarkers to move from a pharmacokinetic-based towards a pharmacodynamic-based monitoring approach for calcineurin inhibitor treatment. Healthy volunteers received a single dose of cyclosporine A (CsA) or placebo, after which whole blood samples were stimulated to measure ex vivo T cell functionality, including proliferation, cytokine production, and activation marker expression. The highest whole blood concentration of CsA was found at 2 h post-dose, which resulted in a strong inhibition of interferon gamma (IFNy) and interleukin-2 (IL-2) production and expression of CD154 and CD71 on T cells. Moreover, the in vitro effect of CsA was studied by incubation of pre-dose whole blood samples with a concentration range of CsA. The average in vitro and ex vivo CsA activity overlapped, making the in vitro dose–effect relationship an interesting method for prediction of post-dose drug effect. The clinical relevance of the results is to be explored in transplantation patients on calcineurin inhibitor treatment.

## 1. Introduction

Therapeutic drug monitoring (TDM) of immunosuppressive treatment is standard of care after kidney transplantation. TDM is mostly used for individualized dosing of calcineurin inhibitors (i.e., tacrolimus and cyclosporin A) since these are known for their large pharmacokinetic intra- and interpatient variability and small therapeutic window. While overexposure to calcineurin inhibitors (CNIs) can lead to adverse events such as nephrotoxicity, neurotoxicity, malignancies, and opportunistic infections, underexposure can result in allograft rejection and loss of the transplanted organ [1]. To prevent toxicity and rejection, patients are being strictly monitored based on whole blood CNI concentrations, especially in the first year after transplantation. Although the incidence of acute rejection has strongly decreased after implementation of CNI therapy with TDM, there are still many patients that experience severe side effects or rejection after several years of treatment, indicating that the current monitoring strategy needs to be improved [2,3].

TDM is based on monitoring pharmacokinetic (PK) variability either by measurement of pre-dose concentrations (C_0_) or ‘limited-sampling’ area under the curve (AUC). More sophisticated PK-based biomarkers have been studied, such as CNI concentrations inside the target cell, but the correlation with clinical outcome is debatable [4,5,6]. None of these parameters, however, do explain the large inter- and intra-patient pharmacodynamic (PD) variability [7]. Hence, new PD-based biomarkers that reflect the immunological status of the patient should be developed to allow monitoring of the individual to the immunosuppressive treatment response and improve personalized dosing. As the first step in this effort, we choose to develop PD-markers for CNIs such as tacrolimus (Tac) and cyclosporin A (CsA), because over the last decade, several promising biomarkers have been identified to monitor drug activity of CNIs. Since both Tac and CsA exert their function by inhibiting the enzyme calcineurin, the most drug-specific biomarker for CNI therapy is measurement of calcineurin enzymatic activity. Several methods to measure calcineurin activity in patients have been studied [8,9,10,11]. However, these methods require cell preparation that results in washing out of the drugs, are laborious, or use radioactive labeling, making them rather cumbersome in clinical practice. Besides enzymatic monitoring of the target enzyme, also general immune markers have been studied as a potential pharmacodynamic monitoring strategy. These include cytokine production [12,13,14], surface marker expression [15], and nuclear factor of activated T cells (NFAT)-mediated gene expression [16], providing insight into the immunosuppressive effect of CNIs at different levels. Although these markers have shown to be informative, none of them has been implemented in clinical practice yet. Overall, the ideal biomarker for TDM correlates well with (prediction of) the occurrence of toxicity and organ rejection and is analytically straightforward. Since transplantation patients receive a combination of immunosuppressive drugs, ideally, these biomarkers reflect the general immune status of the patient rather than drug-specific activity. Because T cells are the main mediators of rejection, and most immunosuppressive therapies aim to inhibit T cell activation, the focus will be on monitoring the functional T cell status. This can be evaluated by cell culture-based assays using whole blood or peripheral blood mononuclear cells (PBMCs), triggered with a T cell agonist. This approach allows quantification of the ex vivo activity of T cell suppressive drugs.

We aimed to develop and select PD biomarkers for future evaluation of the general T cell function of transplantation patients on immunosuppressive therapy, which could eventually be used to support TDM. We focused on whole blood-based biomarkers for evaluation of ex vivo drug activity. As a proof-of-concept, we performed a clinical study on healthy volunteers receiving a single dose of CsA. The aim of this study was to evaluate the PK/PD relationship of CsA, a well-known and widely used T cell-suppressive drug. Various T cell function assays were evaluated in parallel, with the goal of selecting assays for future use in transplantation patients. In addition, CsA concentrations were compared between whole blood, isolated PBMCs and T cells to explore potential differences in CsA PK between matrices.

## 2. Materials and Methods

### 2.1. Study Design

In total, sixteen male and female healthy volunteers were enrolled in this randomized, double-blind, placebo-controlled study. All subjects received a single oral dose of 5 mg/kg CsA (Neoral^®^ capsules, Novartis Pharma, Basel, Switserland), rounded up to the available dosage forms (100 mg and 25 mg Neoral^®^) or placebo. The dosage was based on the recommended daily dose for renal transplant patients receiving cyclosporine as maintenance immunosuppressive therapy (2–6 mg/kg per day in two equal doses). The inclusion criteria were healthy male or female subjects, 18–55 years of age, which gave written informed consent prior to any study-related procedure. The main exclusion criteria were any disease associated with immune system impairment, evidence of any other active or chronic disease, and intake of any nutrients known to modulate CYP enzyme activity. Of the sixteen subjects enrolled in this study, four subjects received placebo, and twelve subjects received active treatment. The subjects were divided into four groups of four subjects and had a total of 3 visits. Both PK and PD samples were taken pre-dose (0 h), 2 h, 3 h, 6 h, 24 h, and 7 days post-dose. This study was approved by the independent medical ethics committee “Medisch Ethische Toetsingscommissie van de Stichting Beoordeling Ethiek Biomedisch Onderzoek” (Assen, the Netherlands) on 4 March 2019, and is registered in the International Clinical Trials Registry Platform (ICTRP) under study number NL7601. The study was performed in compliance with the Dutch laws on drug research in humans.

### 2.2. Whole Blood and Intracellular PK

For measurement of CsA concentrations in whole blood, samples were collected in K2EDTA tubes (Becton Dickinson, Franklin Lakes, NJ, USA) and directly frozen at −80 °C until use. For quantification of intracellular CsA concentrations, PBMCs were isolated using sodium heparin CPT tubes (Becton Dickinson). After isolation, red blood cells were lysed with RBC lysis buffer (Biolegend^®^, San Diego, CA, USA) and PBMCs were resuspended in phosphate-buffered Saline (PBS) (Gibco™, Waltham, MA, USA) and frozen at −80 °C until use. T cells were isolated from sodium heparinized blood (Becton Dickinson) by automated magnetic sorting using RoboSep human T cell isolation kit in combination with RoboSep (Stemcell Technologies Inc., Vancouver, Canada). After a RBC lysis step (Biolegend^®^) the T cells were frozen in PBS (Gibco™) at −80 °C until use.

The quantification of CsA concentrations in whole blood, PBMC and T cell samples was performed by the department of Clinical Pharmacy and Toxicology, Leiden University Medical Center, the Netherlands. CsA concentration in whole blood was quantified using a previously validated LC-MS/MS assay [17,18]. For the quantification of intracellular CsA concentration in PBMCs and T cells, a new method was developed and validated according to EMA bioanalytical method validation guideline. In short, the calibration standards and quality controls were prepared using different stock solutions and cyclosporin-free PBMCs. Stock solutions of cyclosporin A (Supelco^®^, Sigma Aldrich, St. Louis, MO, USA) and cyclosporin A-D12 (Alsachim, Illkirch-Graffenstaden, France) (1 mg mL^−1^) were prepared in acetonitrile (Merck, Darmstadt Germany) and stored at −20 °C. Substock solutions of 10 mg/L were prepared by diluting the stock solution in acetonitrile. Calibration standards were 0.1, 0.2, 0.5, 1, 5, 10, 20, 50, and 100 ug/L. The lower limit of quantification was set at 0.1 ug/L. QC’s low, medium, and high of 0.5, 5, and 50 ug/L, respectively, were used in every analytical run. All were prepared by diluting the working solution with cyclosporin-free PBMC’s. Samples that were expected to exceed the calibration curve were diluted 1:1. 100 μL of sample was mixed with 20 μL of internal standard solution (200 μg/L), 40 μL of 0.1 M zinc sulfate, and 100 μL acetonitrile and vortexed for 5 min at 2000 rpm. Subsequently, the samples were centrifuged for 5 min at 1300 rpm, and a 200 μL aliquot of the supernatant was transferred to an autosampler insert vial. A volume of 40 μL was injected into the LC system. All samples were analyzed on a Quantiva UPLC-MS/MS system, consisting of a Dionex Ultimate 3000 series UHPLC system, coupled to a TSQ Quantiva triple stage quadrupole mass spectrometer, all from ThermoFisher Scientific (Waltham, MA, USA). Data were acquired and processed using ThermoFisher Scientific Chromeleon software version 7.2. Chromatographic separation was achieved using an Acquity UPLC BEH C18 1.7 µm 2.1 × 50 mm column, coupled to an Acquity UPLC BEH C18 1.7 µm 2.1 × 5 mm precolumn, both from Waters. The column heater was set to 65 °C. Gradient elution was performed with a mobile phase consisting of a mixture of 0.1% formic acid and 2 mM ammonium in water (eluent A) or methanol (eluent B). Ultrapure water was produced onsite using a PURELAB^®^ Flex purification system from ELGA LabWater (Lane End, UK). The elution gradient (eluent A%/eluent B%) was 80/20 from initiation to 1.5 min, 98/2 from 1.5 min to 4.2 min, and 80/20 from 4.2 to completion of the run at 5.5 min, with a constant flow and pressure of 0.4 mL/min and 350 bar, respectively. The MS instrument was operated in the ESI+ mode, electrospray voltage was 4600 V, capillary temperature was 300 °C, and vaporizer temperature was 350 °C. Sheath, auxiliary, and sweep gas flow rates were set at 18.5, 9.3, and 3.3 arbitrary units, respectively.

The following mass transitions were used for multiple reaction monitoring acquisition (m/z): cyclosporine A 1202.8 → 1184.9, [^2^H_12_]-cyclosporine A 1214.8 → 1196.9. All analytical validation parameters were in accordance with the EMA bioanalytical method validation guideline.

### 2.3. Whole Blood Culture and PD Assessments

For all PD assessments, sodium heparinized whole blood (Becton Dickinson) was incubated at 37 °C, 5% CO_2_, and stimulated with 10 μg/mL phytohemagglutinin (PHA) (Merck). At the pre-dose time point, the in vitro CsA concentration–effect relationship for each individual subject was studied by incubating whole blood samples with a concentration of 10, 3.3, 1.1, 0.36, and 0.12 μg/L CsA (Merck). To study the immunosuppressive effect of CsA ex vivo, all whole blood samples post-dose were incubated with PHA only.

For analysis of T cell activation marker expression, the whole blood samples were incubated for 6 h. Red blood cells were lysed using RBC lysis buffer (Biolegend^®^), and the samples were stained for flow cytometry analysis with anti-CD3-Viogreen, anti-CD69-APCVio770, anti-CD95-PEVio770, anti-CD25-PE, anti-CD71-FITC, and anti-CD154-VioBlue (Miltenyi Biotec, Köln, Germany). Propidium iodide (Miltenyi Biotec) was added, and samples were measured using a MACSQuant 10 analyser (Miltenyi Biotec). Cytokine production was analysed after 24 h incubation, and supernatant was collected and stored at −80 °C until analysis. IFNγ and IL-2 concentrations were measured by the Meso Scale Discovery Vplex-2 method by Ardena Bioanalytical Laboratory in Assen, the Netherlands.

To analyse T cell proliferation, the whole blood samples were incubated for 48 h with PHA and 20 µM of the labelled nucleoside analogue EdU (5-ethynyl-2’-deoxyuridine) (Thermo Fisher Scientific, Waltham, MA, USA). After red blood cell lysis, the EdU assay was continued according to the manufacturer’s instructions. The cells were stained with anti-CD3-VioGreen (Miltenyi Biotec) and viability dye eFluor780 (Thermo Fischer Scientific) for flow cytometry analysis and were analysed using MACSQuant 10 analyser.

### 2.4. Data Analysis

Analysis of flow cytometry data was performed with Flowlogic software (Inivai Technologies, Mentone VIC, Australia). The gating strategy is shown in Figure S1. Data of all plots are presented as mean value ± standard deviation (SD). No formal power analysis was performed for this explorative study with new cell-based biomarkers. For that reason, no formal statistics were applied to discriminate between active and placebo treatment. IC50s of in vitro CsA activity was calculated using Graphpad Prism 9.4 (GraphPad software Inc., San Diego, CA, USA).

## 3. Results

### 3.1. Subject Characteristics and Safety

A total of 12 subjects received a single dose of Neoral (CsA), and 4 subjects received placebo. The baseline characteristics of the 16 healthy volunteers are summarized in Table 1. A total of 35 Treatment Emergent Adverse Events (TEAE) occurred during the study, of which 32 in the Neoral (CsA) group and 3 in the placebo group. All TEAE were mild in severity, transient, and resolved spontaneously (summary in Table 1). No clinically relevant changes in blood chemistry, hematology, urinalysis, vital signs, or ECG were identified during the study.

### 3.2. Pharmacokinetics

**Figure 1:**
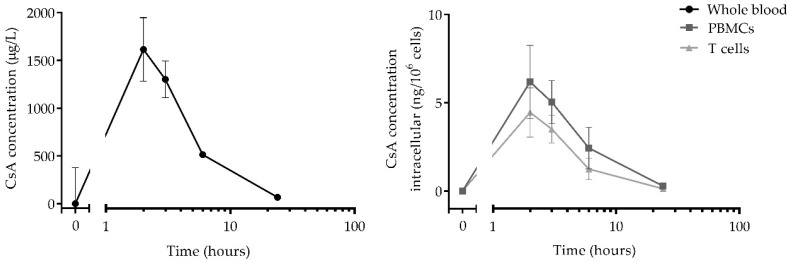
Mean concentration of CsA in whole blood, PBMCs, and T cells. Samples were taken at 0 h, 2 h, 3 h, 6 h, and 24 h post-dose.

Concentrations of CsA were measured in three different matrices: whole blood, PBMCs, and T cells. All PK profiles are shown in Figure 1. The CsA levels in whole blood were highest (1615.3 ± 374 µg/L) at 2 h post-dose and almost returned to baseline levels at 24 h post-dose. The intracellular PK profiles followed a similar profile as CsA levels in whole blood, with peak concentrations of 6.2 ng/10^6^ cells (±2.0 ng/10^6^ cells) in PBMCs and 4.4 ng/10^6^ cells (±1.4 ng/10^6^ cells) in T cells at 2 h post-dose. Moreover, the CsA concentration in T cells was, on average, 70% of the concentration in PBMCs.

### 3.3. CsA Strongly Inhibits PD Markers Post-Dose

**Figure 2:**
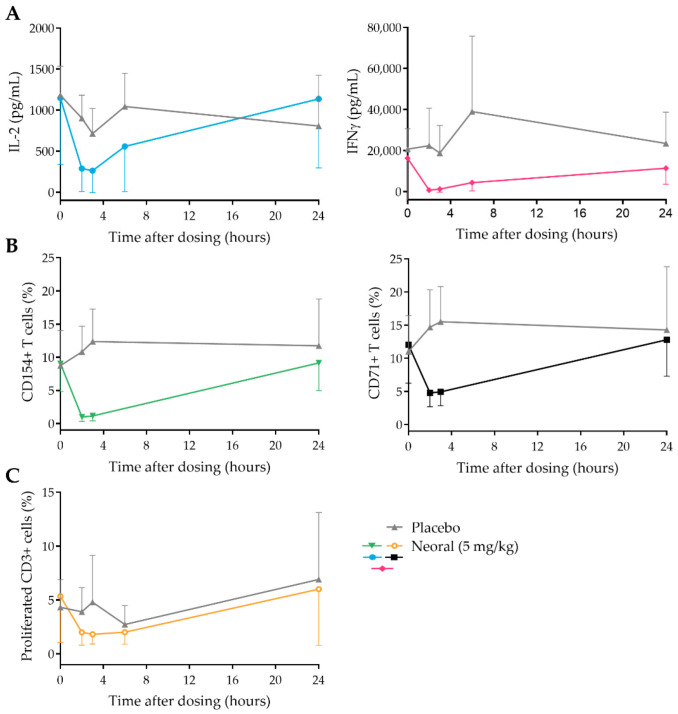
Ex vivo cytokine production (**A**), T cell activation marker expression (**B**), and T cell proliferation (**C**) after a single dose of 5 mg/kg Neoral (colored lines) or placebo (grey lines). Samples were taken at 0 h, 2 h, 3 h, 6 h, and 24 h post-dose.

To study the immunosuppressive effect of CsA administration on the selected PD markers, whole blood samples taken at 0, 2, 3, 6, and 24 h post-dose were stimulated with PHA. After incubation, ex vivo cytokine production (IL-2 and IFNy), T cell activation marker expression (CD71, CD154, CD69, and CD25), and T cell proliferation were measured. All markers, except for CD69 and CD25 (Figure S2), clearly decreased at 2- and 3-h post-dose and returned to baseline at 24 h in the CsA-treated group. The largest CsA effect (compared to baseline and placebo) was found for cytokine production and T cell activation markers (Figure 2A,B). Although the difference is small, also for T cell proliferation, it was possible to discriminate between the CsA and placebo group (Figure 2C). Interestingly, the level of inhibition of all PD markers was similar at the 2- and 3-h time points, while the CsA concentrations seemed to differ at these time points (Figure 1). This could indicate that the CsA concentrations 2- and 3-h post-dose both result in maximum inhibition of the PD markers or that the duration of the PD effect of CsA is longer than the presence of CsA in the cells.

### 3.4. In Vitro Concentration–Effect Relationship of CsA

**Figure 3:**
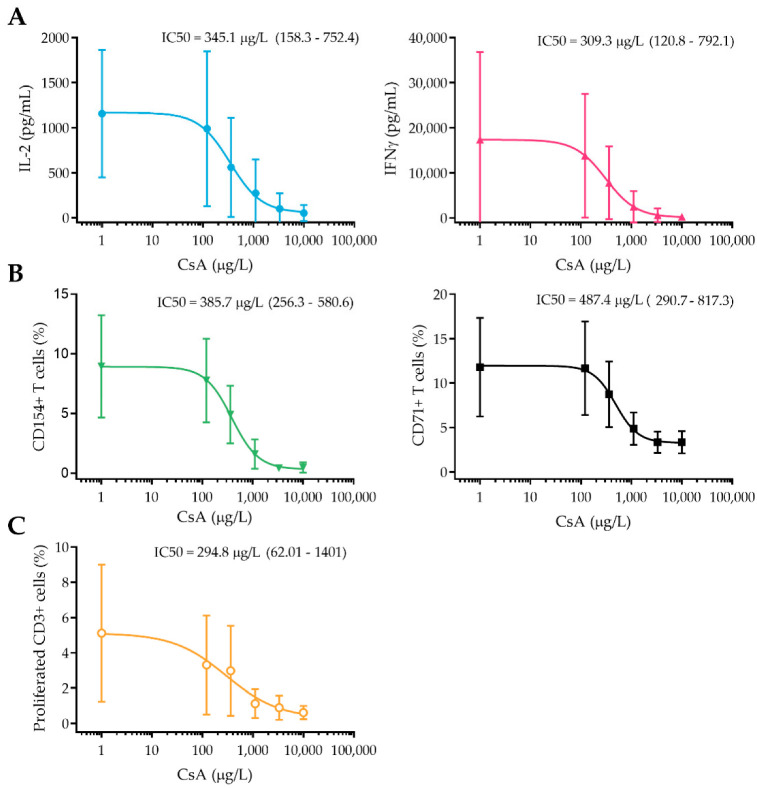
In vitro CsA activity on cytokine production (**A**), T cell activation marker expression (**B**), and T cell proliferation (**C**). All whole blood samples were taken pre-dose, stimulated with PHA, and incubated with a concentration range of CsA (10,000, 3300, 1100, 360, 120 µg/L). Absolute data points (±SD) and logistic regression model are plotted.

Besides monitoring the ex vivo drug activity, also the in vitro concentration–effect relationship of CsA was studied. At timepoint 0 h, whole blood samples of each subject were stimulated with PHA in the presence of a concentration range of CsA, after which in vitro cytokine production, T cell activation marker expression, and T cell proliferation were measured. In Figure 3 in vitro concentration-response relationship of CsA for all PD markers is shown. IL-2 and IFNγ production, together with CD154 expression were most strongly affected by CsA (IC50 of 345, 309, and 385 µg/L, respectively, with 95% CI of 158–752, 120–792, and 256–581), reaching complete inhibition at 3300 µg/L CsA. For CD71 expression, the IC50 was slightly higher than for the other markers (487 µg/L), and its expression could not be completely inhibited, not even at the highest concentration of CsA. T cell proliferation, on the other hand, showed the strongest dose–effect relationship with an IC50 of 294 µg/L but was more variable between subjects (IC50 95% CI of 62–1401). Absolute in vitro data, without the logistic regression model, are shown in Figure S3.

### 3.5. Correlation of In Vitro and Ex Vivo Drug Effect

**Figure 4:**
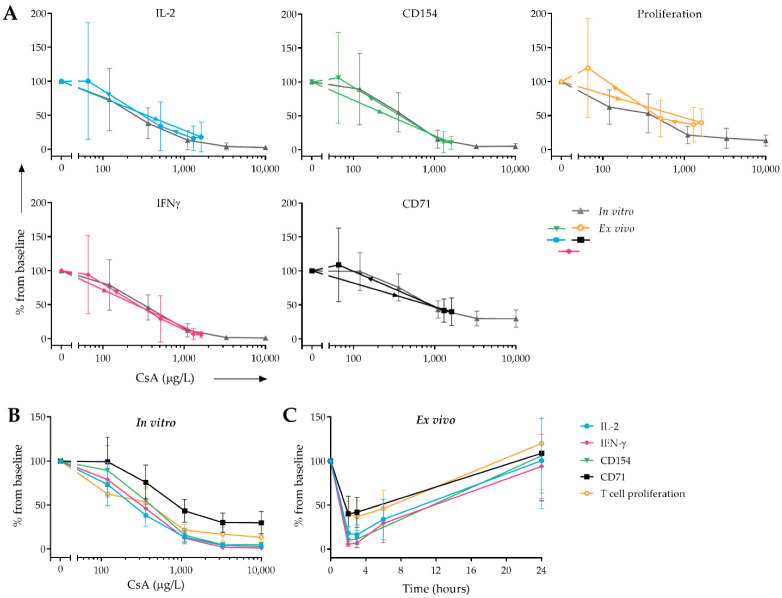
Overlay of in vitro and ex vivo CsA effect on cytokine production, T cell activation marker expression, and T cell proliferation (**A**). In grey, the in vitro concentration–effect relationship of CsA for each individual PD marker is shown. In color (green, yellow, blue, black, and pink), the ex vivo effect of CsA on each PD marker is shown. Arrows indicate the time course of the samples (0 h, 2 h, 3 h, 6 h, and 24 h). In vitro (**B**) and ex vivo (**C**) effect of CsA on selected PD markers (IL-2, IFNγ, CD154, CD71, and T cell proliferation), expressed as percentage from baseline.

To study the association between the in vitro concentration–effect relationship of CsA (as shown in Figure 3) and the ex vivo CsA effect post-dose (as shown in Figure 2), an overlap of mean in vitro and ex vivo drug effect is plotted in Figure 4A. There is a clear overlap between both plots, indicating that the in vitro dose–effect relationship seemed a good predictor of the ex vivo drug effect for all PD markers.

In Figure 4B all PD markers are expressed as percentage from baseline. With a maximum inhibition of >95% in vitro and >80% ex vivo, the strongest CsA-dependent inhibition was found for IFNγ production, IL-2 production, and CD154 expression. CD71 expression and T cell proliferation showed a smaller CsA-dependent decrease (inhibition of 70% and 86.7% in vitro and 60.1% and 63.3% ex vivo, respectively), but still were clearly suppressed by CsA.

## 4. Discussion

Calcineurin inhibitors (i.e., tacrolimus and cyclosporin A) have a large pharmacokinetic variability and small therapeutic window. To optimize dosing regimens, therapeutic drug monitoring (TDM) of calcineurin inhibitors is standard of care after solid organ transplantation. However, this PK-based monitoring strategy apparently provides limited information as transplantation patients still experience rejection of the transplanted organ or severe side effects after several years of treatment [19]. In this study, we therefore aimed to identify PD biomarkers that reflect T cell functionality and activity of immunosuppressive medication for future PD-focused TDM of calcineurin inhibitors in transplantation patients.

We performed a study on healthy volunteers receiving a single dose of cyclosporin A, after which drug concentrations were measured in whole blood, PBMCs, and T cells. To explore if drug concentrations in the target cell (PBMC and T cells) are more informative as a readout measure for TDM compared to the currently used whole blood concentrations, the PK between these three matrices were compared. The highest whole blood concentration of CsA was detected 2 h after drug administration, returning to baseline at 24 h post-dose, which is in line with previously reported PK profiles of CsA in healthy volunteers and patients [20,21]. The intracellular concentrations measured in PBMCs and T cells showed a comparable pharmacokinetic profile, peaking at 2 h post-dose and returning to baseline at approximately 24 h, similar to whole blood. Although for tacrolimus, there is an ongoing debate about the relevance of intracellular drug concentrations compared to whole blood concentrations [4], there is limited literature available for CsA [22]. Based on our results, we conclude that intracellular CsA concentrations do not carry additional value over whole blood concentrations, which is in line with what we previously found for tacrolimus [23]. The current whole blood-based TDM for CNIs seems to be a good representation of the concentrations found in the target cell.

Despite the good correlation between whole blood and intracellular concentrations, concentration-based TDM of calcineurin inhibitors is known to be suboptimal. We aimed to identify biomarkers that reflect the general immune status of the transplantation patient and that could be used for monitoring calcineurin inhibitor activity at a cellular level. Since T cells are the main mediators of rejection, most immunosuppressive therapies, including CsA, aim to inhibit T cell activation. CsA inhibits the enzyme calcineurin, thereby preventing NFAT activation and subsequent anti- and pro-inflammatory gene expression, including cytokines, chemokines, growth factors, and metabolic regulators [24]. We evaluated various T cell function assays in parallel, with the goal of selecting assays for future use in transplantation patients. We stimulated whole blood with PHA to drive this T cell activation and evaluate ex vivo CsA activity at three different levels: cytokine production, T cell activation marker expression, and proliferation.

From a physiological point of view, IL-2 production is the most interesting PD biomarker. It is one of the first cytokines to be produced upon T cell activation, mediated by NFAT, and an important inducer of anti- and pro-inflammatory gene expression [25]. We found that whole-blood stimulated IL-2 production was strongly reduced (with 82% ± 22% compared to baseline, respectively) in CsA-treated subjects compared to placebo at 2 h post CsA administration. In vitro, a strong dose–effect relationship for CsA was also found, correlating with the inhibitory CsA effect that was measured ex vivo. For IFNγ a similar reduction was found ex vivo (inhibition of 94% ± 5% compared to baseline at 2 h post-dose) and in vitro (maximum inhibition of 99% ± 1%). Although IFNγ is not only produced by T cells, it is a pro-inflammatory cytokine that is essential in the innate and adaptive immune response and strongly affects T cell function.

While cytokine production is detectable a few hours after T cell activation, surface activation markers can be expressed within minutes after stimulation of the T cell receptor (TCR). In this study, we focused on four different surface markers as potential PD readout measures, two immediate early (CD69 and CD25) and two mid-early T cell activation markers (CD71 and CD154). CD69 is a type II C-lectin receptor, and CD25 is the alpha chain of the IL-2 receptor, both are rapidly expressed after T cell activation and are important for proliferation and activation. Although CD69 and CD25 are strongly associated with T cell activation [26,27], with our experimental setup, no effect of CsA on these markers was found, neither in vitro nor ex vivo. The mid early activation marker CD154 (CD40 ligand) and CD71 (transferrin receptor 1) showed a strongly decreased expression after CsA administration (of 90 ± 9% and 60 ± 20%, respectively), which corresponded to the inhibitory effect of CsA that was found in vitro. CD40 ligand is a co-stimulatory molecule that interacts with CD40 and is primarily expressed by T helper cells. Inhibition of this interaction is currently studied as potential anti-rejection therapy for transplantation patients [28]. Transferrin receptor 1 is a marker that is upregulated after activation to increase the iron uptake of the activated T cell, which is essential for proliferation and known to be dependent on the presence of IL-2 [29].

The purpose of the increased cytokine production and expression of activation markers after TCR activation is to induce proliferation and differentiation of T cells and thereby start the adaptive immune response. To investigate whether a PD marker more distal to TCR stimulation could be a relevant readout measure for CsA activity, PHA-induced T cell proliferation was measured. Administration of CsA to healthy volunteers resulted in a strong inhibition of T cell proliferation, which is not surprising given the strong inhibition of IL-2, an important inducer of T cell proliferation. Overall, we conclude that IL-2 and IFNγ production, CD154 and CD71 expression, and T cell proliferation are good biomarkers to monitor the immunosuppressive effect of CsA. T cell proliferation is the most laborious readout measure with the longest incubation times, while cytokine production and activation marker expression assays are simpler to execute and may be easier to standardize for clinical practice. Together with our findings that IFNγ, IL-2, and CD154 showed the strongest dose-response relationship with the smallest variation, these readout measures appear to be most suitable for immunomonitoring of CNI in clinical practice.

When comparing the in vitro dose–effect relationship of CsA with our ex vivo results of the selected markers, there is a clear correlation. At the individual level, the overlap between in vitro and ex vivo plots can vary, but the mean in vitro dose-response curve seems to be a good predictor for the ex vivo inhibitory CsA effect observed after dosing. While whole blood and intracellular CsA concentrations started decreasing at 3 h post-dose (from 1615 µg/L at 2 h to 1300 µg/L at 3 h), this was not reflected at the level of cellular CsA activity. At three hours after administration, all PD endpoints still showed a maximal inhibitory effect of CsA. This suggests that at a concentration of 1300 ug/L the maximum possible inhibition of these markers was reached, which is in line with our in vitro data, where maximum inhibition of all markers is reached at 1100 µg/L CsA. As the CsA target ranges are trough level (C0) of 100–200 µg/L, and a peak level (C2) of 700–900 µg/L CsA in stable renal transplantation patients [30], it is likely that these patients have varying levels of immune suppression during the day and never reach maximum inhibition of T cell function. Moreover, the in vitro concentration–effect curves of all cellular PD markers have a sigmoidal shape, indicating that the relationship between PK and PD is not a linear but a logistic regression. This suggests that measurement of PD markers, such as cytokine production and T cell activation marker expression, provides more insight into the immunosuppressive state of a patient than the measurement of whole blood drug concentrations. This relationship will be further studied using a PK/PD modelling approach.

In conclusion, we conducted a healthy volunteer study to characterize and select pharmacodynamic markers for monitoring CsA activity and assessment of functional T cell status. We showed that pharmacokinetic profiles for CsA were well comparable between whole blood, PBMCs and T cells, underlining the limited added value of monitoring of intracellular CsA concentrations. We identified several markers (IL-2, IFNγ, CD71, CD154, T cell proliferation) that convincingly showed the immunosuppressive effects of CsA. Moreover, the mean in vitro CsA concentration–effect relationship for these markers overlapped with the ex vivo drug effect. To evaluate the potential additional clinical value of these PD markers comparted to the current PK-based TDM strategy, a clinical study in renal transplantation patients is planned.

## Figures and Tables

**Table 1:** Baseline subject characteristics and treatment emergent adverse events by treatment. All TEAEs were coded using the Medical Dictionary for Regulatory Activities (MedDRA) version 24.1. The grey rows depict the system organ classes. Multiple TEAEs could be reported by the same subject.

Subject characteristics	5 mg/kg Neoral (*n* = 12)		Placebo (*n* = 4)	
Age (range)	28.9 (21–52)		25.5 (22–28)	
Gender (female/male)	4/8		2/2	
BMI (kg/m^2^), mean (range)	23.3 (19–26.4)		24.0 (21.5–27.5)	
**System Organ Class/Preferred term**	**Events**	**Subjects (%)**	**Events**	**Subjects (%)**
Any events	32	11 (91.7)	3	3 (75.0)
Gastrointestinal disorders	5	5 (41.7)	-	-
Abdominal pain	1	1 (8.3)	-	-
Faeces pale	1	1 (8.3)	-	-
Nausea	2	3 (25.0)	-	-
General disorders and administration site conditions	18	7 (58.3)	-	-
Burning sensation	1	1 (8.3)	-	-
Fatigue	5	3 (25.0)	-	-
Feeling cold	1	1 (8.3)	-	-
Feeling hot	6	3 (25.0)	-	-
Hyperhidrosis	1	1 (8.3)	-	-
Peripheral coldness	4	3 (25.0)	-	-
Infections and infestations	1	1 (8.3%)	-	-
Candida infection	1	1 (8.3%)	-	-
Nervous system disorders	6	6 (50.0)	3	3 (75.0)
Dizziness	-	-	1	1 (25.0)
Headache	5	5 (41.7)	2	2 (50.0)
Somnolence	1	1 (8.3)	-	-
Renal and urinary disorders	1	1 (8.3)	-	-
Chromaturia	1	1 (8.3)	-	-
Respiratory, thoracic, and mediastinal disorders	1	1 (8.3)	-	-
Nasopharyngitis	1	1 (8.3)	-	-

## Data Availability

The data presented in this study are available on request from the corresponding author.

## Supplementary Materials

The following supporting information can be downloaded at: https://www.mdpi.com/article/10.3390/pharmaceutics14091958/s1, Figure S1: Gating strategy of T cell activation marker expression and T cell proliferation; Figure S2: Ex vivo T cell activation marker expression after a single dose of 5 mg/kg Neoral or placebo; Figure S3: In vitro dose effect of CsA on cytokine production, T cell activation marker expression and T cell proliferation with IC50.
