# Supplementary material for: Monitoring of Ex Vivo Cyclosporin a Activity in Healthy Volunteers Using T Cell Function Assays in Relation to Whole Blood and Cellular Pharmacokinetics
Source: Pharmaceutics. 2022 Sep 16;14(9):1958. doi: 10.3390/pharmaceutics14091958 (PMC9503885; doi:10.3390/pharmaceutics14091958)
Supplement: Supplementary file 1 [file pharmaceutics-14-01958-s001.zip › CHDR1860_Supplemental material.pdf]

Supplementary material

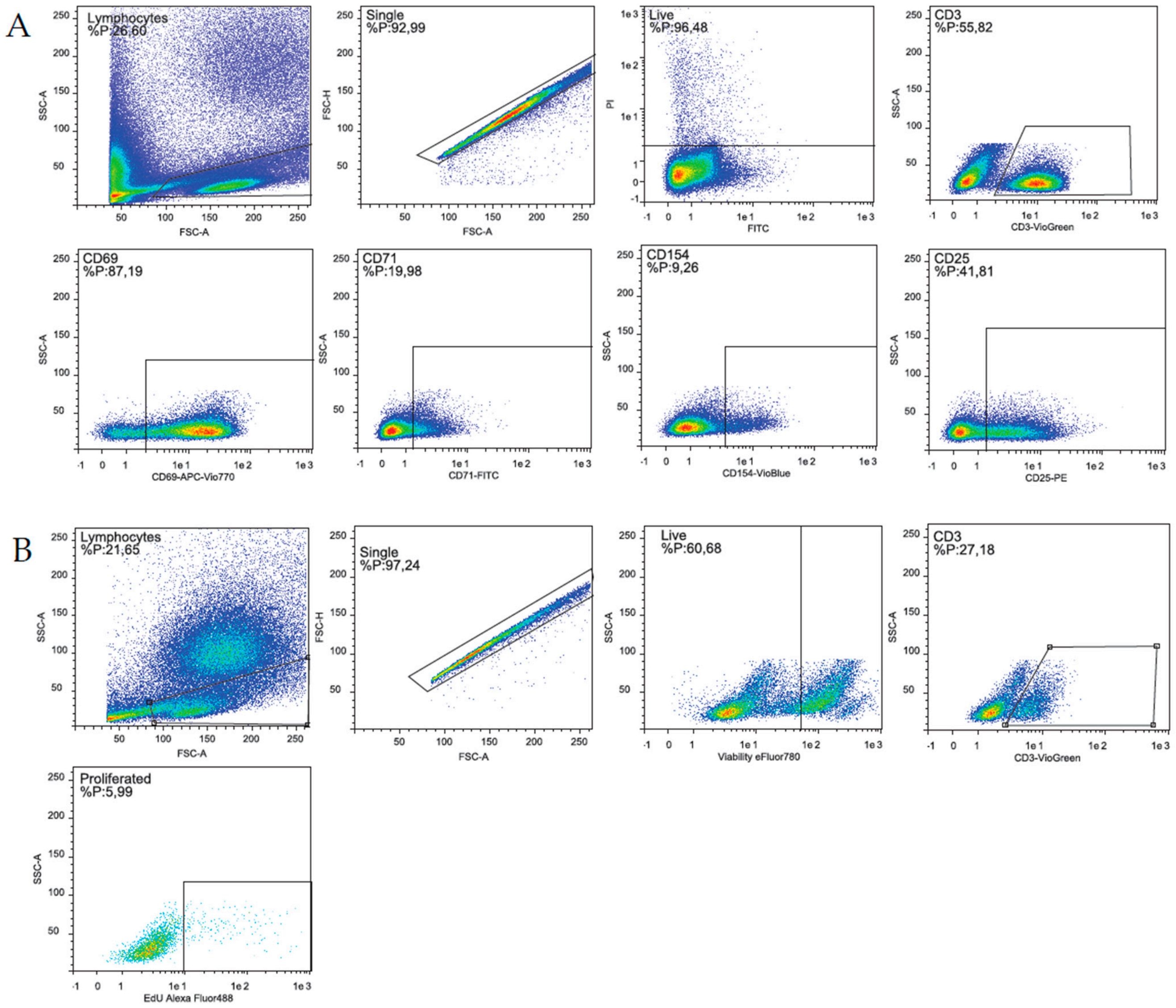

Figure S1: Gating strategy of T cell activation marker expression **(A)** and T cell proliferation **(B)**

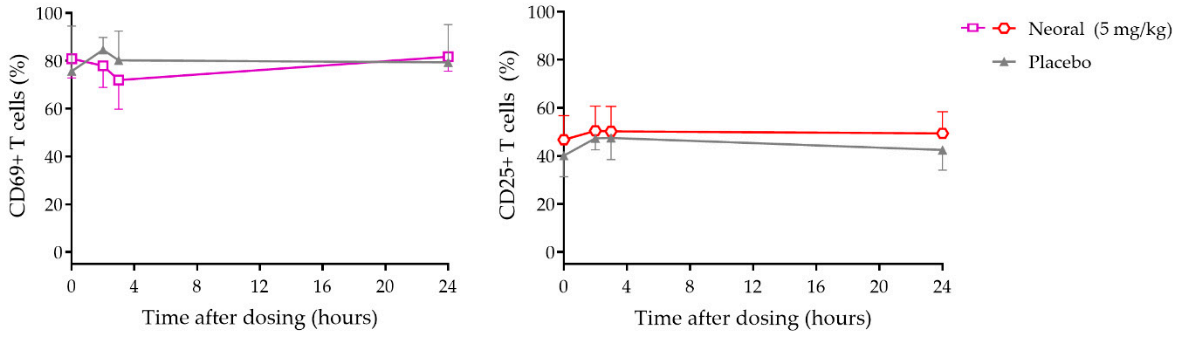

Figure S2: *Ex vivo* T cell activation marker expression after a single dose of 5 mg/kg Neoral or placebo

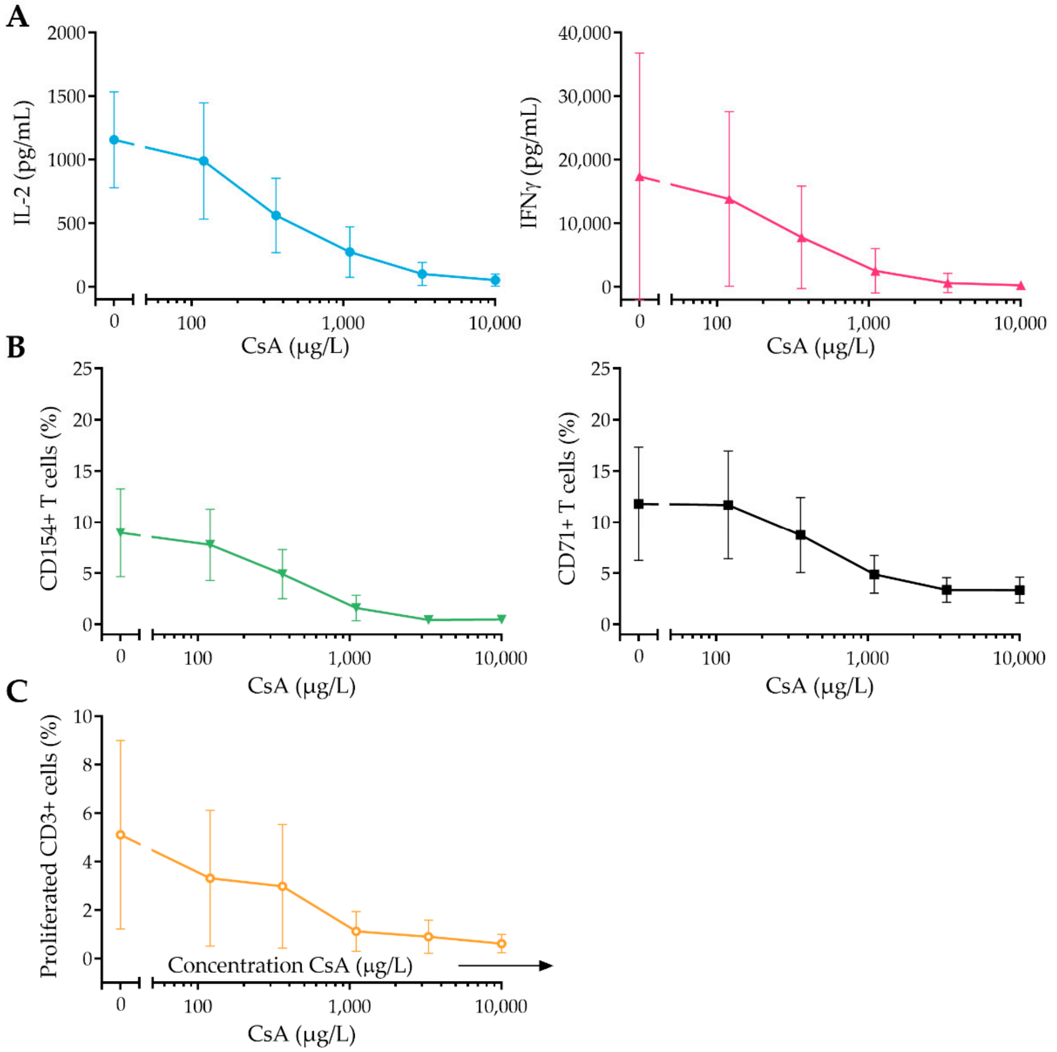

Figure S3: *In vitro* dose effect of CsA on cytokine production, T cell activation marker expression and T cell proliferation.
